# Non-alcoholic fatty liver disease: pathophysiological concepts and treatment options

**DOI:** 10.1093/cvr/cvad095

**Published:** 2023-06-26

**Authors:** Christoph Grander, Felix Grabherr, Herbert Tilg

**Affiliations:** Department of Internal Medicine I, Gastroenterology, Hepatology, Endocrinology & Metabolism, Medical University Innsbruck, Anichstrasse 35, Innsbruck 6020, Austria; Department of Internal Medicine I, Gastroenterology, Hepatology, Endocrinology & Metabolism, Medical University Innsbruck, Anichstrasse 35, Innsbruck 6020, Austria; Department of Internal Medicine I, Gastroenterology, Hepatology, Endocrinology & Metabolism, Medical University Innsbruck, Anichstrasse 35, Innsbruck 6020, Austria

**Keywords:** Non-alcoholic liver disease, NAFLD, Cardio vascular disease, Treatment, Pathophysiology, Diagnosis

## Abstract

The prevalence of non-alcoholic fatty liver disease (NAFLD) is continually increasing due to the global obesity epidemic. NAFLD comprises a systemic metabolic disease accompanied frequently by insulin resistance and hepatic and systemic inflammation. Whereas simple hepatic steatosis is the most common disease manifestation, a more progressive disease course characterized by liver fibrosis and inflammation (i.e. non-alcoholic steatohepatitis) is present in 10–20% of affected individuals. NAFLD furthermore progresses in a substantial number of patients towards liver cirrhosis and hepatocellular carcinoma. Whereas this disease now affects almost 25% of the world’s population and is mainly observed in obesity and type 2 diabetes, NAFLD also affects lean individuals. Pathophysiology involves lipotoxicity, hepatic immune disturbances accompanied by hepatic insulin resistance, a gut dysbiosis, and commonly hepatic and systemic insulin resistance defining this disorder a prototypic systemic metabolic disorder. Not surprisingly many affected patients have other disease manifestations, and indeed cardiovascular disease, chronic kidney disease, and extrahepatic malignancies are all contributing substantially to patient outcome. Weight loss and lifestyle change reflect the cornerstone of treatment, and several medical treatment options are currently under investigation. The most promising treatment strategies include glucagon-like peptide 1 receptor antagonists, sodium–glucose transporter 2 inhibitors, Fibroblast Growth Factor analogues, Farnesoid X receptor agonists, and peroxisome proliferator–activated receptor agonists. Here, we review epidemiology, pathophysiology, and therapeutic options for NAFLD.

## Graphical Abstract

**Graphical Abstract:**
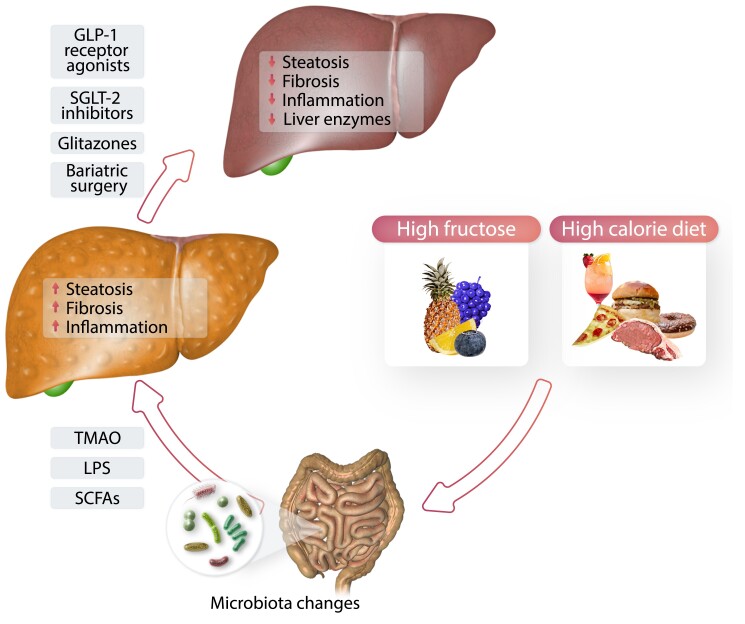

## 1. Introduction

The prevalence of non-alcoholic fatty liver disease (NAFLD) is increasing globally and is expected to become the leading cause of liver transplantation by 2030, with expanding costs for the healthcare systems.^1^ NAFLD comprises a large spectrum of disease entities from simple hepatic steatosis to non-alcoholic steatohepatitis (NASH), liver fibrosis, cirrhosis, and hepatocellular carcinoma (HCC). NAFLD has an estimated prevalence of 25% in the general population^2^ with even higher prevalence in populations with metabolic diseases. Patients with type 2 diabetes (T2D) exhibit a NAFLD prevalence up to 75%,^3^ and severely obese patients show prevalence rates of even 90%.^4,5^ A recently published cross-sectional population-based study estimated the prevalence of advanced fibrosis in Germany at 1%.^6^ NAFLD has evolved as a prototypic systemic disease in the past decade, and importantly extrahepatic diseases such as cardiovascular disease (CVD) or extrahepatic malignancies are the major contributors to mortality in this population.^7–9^ The stringent associations of NAFLD with its mortality-driving co-morbidities are not well understood but may include various aspects including continuous low-grade inflammation observed in NAFLD.^10,11^

NAFLD is defined by an excessive hepatic fat accumulation, associated with insulin resistance (IR) and evidence of steatosis based on imaging techniques or histology. Furthermore, secondary causes of hepatic steatosis like alcohol consumption (>30 g for men and >20 g for women) need to be ruled out.^12^ In 2020, Eslam *et al*. proposed alternative diagnostic criteria for NAFLD and also suggested an alternative term: metabolic associated fatty liver disease (MAFLD). Instead of the exclusion of alcohol use, ‘positive criteria’ were defined. MAFLD is present in patients with observed hepatic steatosis [as detected by ultrasound, computed tomography, magnetic resonance spectroscopy, or controlled attenuation parameter (CAP, FibroScan)] and overweight [body mass index (BMI) ≥25 kg/m^2^ in Caucasians or BMI ≥23 kg/m^2^ in Asians] or T2D. In lean/normal weight patients, two of the following factors need to be present in addition to hepatic steatosis: increased waist circumference, arterial hypertension, elevated triglycerides, decreased plasma high-density lipoprotein cholesterol (HDL-c), pre-diabetes, elevated homoeostasis model assessment of insulin resistance (HOMAR) score, or increased plasma high-sensitivity C-reactive protein. A controversial discussion has evolved around this new, inclusive, diagnostic definition.^13^ Some studies indicate that using the new definition rather than the old one results in a higher detection of patients suffering from liver disease,^14^ which would result in better/optimized patient care. On the other side, the new definition criteria fail to include NASH, the aggressive form of NAFLD including inflammation and liver injury. However, there are still some unmet needs for this new definition and therefore we will use the term NAFLD throughout this review. Historically, NAFLD was and still is associated mainly with obese individuals; however, in the past years, the recognition of *lean NAFLD* as an entity of NAFLD has emerged. Recently, a new clinical practice guideline has been published,^15^ which supports the importance of diagnosing lean NAFLD patients [e.g. NAFLD and BMI <25 (non-Asian) and <23 kg/m^2^ (Asian)] and also identifies co-morbidities such as T2D, dyslipidaemia, hypertension, and fibrotic changes of the liver. However, a screening of otherwise healthy people for lean NAFLD is currently not recommended but should be considered in T2D patients older than 40.^15^

### 1.1 Pathophysiology of NAFLD

The pathophysiology of NAFLD is complex and heterogenous, already illustrated by the fact that NAFLD comprises a clinical spectrum from simple steatosis to cirrhosis as end stage of liver disease. Many different factors are involved in inducing metabolic associated changes in the liver. An overconsumption of nutrients can lead to dysbiosis in the gastrointestinal tract; further a translocation of microbial-associated molecular patterns to the liver via the portal vein and into the systemic circulation via an increased permeability of the intestinal barrier can induce pro-inflammatory reactions in the liver. On the other side, certain dietary components can also directly trigger relevant disease mechanisms in liver tissue.^16–18^

#### 1.1.1 Lipotoxicity

One of the characterizing features of NAFLD on histopathological level is the accumulation of lipid droplets in hepatocytes.^19^ Therefore, a possible disease-driving role of lipids and lipid-derived compounds has been assumed since a long time. Harbouring a SNP in *PNPLA3* (rs738409, I148M) increases the genetic susceptibility towards the development of NAFLD.^20^ This protein is in close proximity to lipid droplets within the hepatocyte.^21,22^ The I148m alteration in PNPLA3 leads to an altered remodelling of fatty acids in the hepatocytes; further this variant leads to an accumulation of PNPLA3 on lipid droplets, as the degradation of the protein via the ubiquitination pathway is reduced compared with the wild-type protein.^21–23^ The knockdown of the protein resolves steatosis in an experimental murine steatosis model, indicating that a knockout/inhibition of the enzyme would be a possible treatment target.^22^ Recently, the germinal centre kinase III (GCKIII) kinases Mammalian sterile 20-like (MST)-3 and MST4 were described to correlate positively with increased histopathological disease severity in NAFLD patients.^24–27^ These kinases associate with lipid droplets within the hepatocyte^26–28^ and control lipid-induced metabolic stress in hepatocytes. siRNA silencing experiments in human hepatocytes showed that reduced levels of MST3, MST4, and serine/threonine-protein kinase 24 (STK24) led to a decrease in triacylglycerol (TAG) synthesis and thereby to a reduction in lipid droplet formation. Further, it seems that these three proteins inhibit β-oxidation and thereby drive oxidative stress, which is a key pathomechanism of lipotoxicity in NAFLD.^24–29^

**Figure 1:**
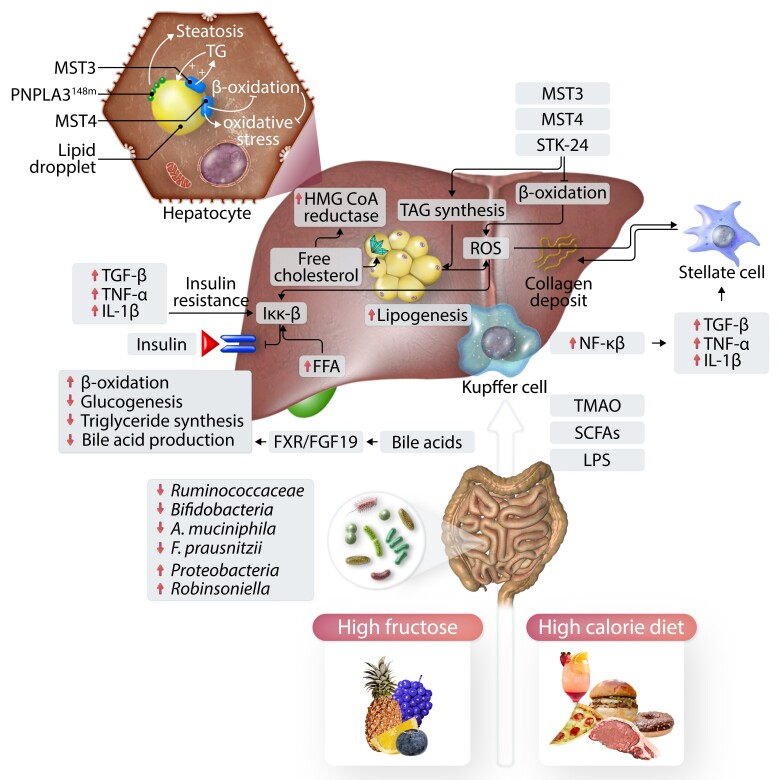
The pathogenesis of non-alcoholic fatty liver disease (NAFLD) is multifactorial. Diet and dietary components effect intestinal microbiota and influence hepatic inflammation and steatosis. FFAs, reactive oxygen species (ROS), and low-grade inflammation mediate insulin resistance by altering IKK-β. Increased lipogenesis and free cholesterol further add cellular stress (lipotoxicity). LPS activates hepatic Kupffer cells to produce pro-inflammatory cytokines. SCFAs and TMAO are metabolites derived from diet components through the intestinal microbiota. Together, different mechanisms induce inflammation (e.g. production of pro-inflammatory cytokines), which activates stellate cells to produce collagen and induce fibrogenesis. FFA, free fatty acids; FXR, Farnesoid X receptor; IKK-β, inhibitor of nuclear factor kappa-B kinase subunit beta; IL-1b, interleukin 1 beta; LPS, lipopolysaccharide; MST-3 and MST-4, GCKIII kinases Mammalian sterile 20-like 3 and 4; NF-κB, nuclear factor of activated B cells; ROS, reactive oxygen species; SCFA, Short chain fatty acids; TAG, triacylglycerol; TGFβ, transforming growth factor beta; TMAO, trimethylamine-N-oxide; TNF-α, tumour necrosis factor alpha.

Another compound with possible lipotoxic functions is free cholesterol.^30^ The expression of 3-hydroxy-3-methylglutaryl (HMG) CoA reductase, the rate-limiting enzyme in cholesterol synthesis, is up-regulated in liver tissue of NAFLD patients compared with lean and obese controls.^31^ This up-regulation was paralleled by a dephosphorylation, thus more activation of HMG CoA reductase and an increase in free cholesterol synthesis.^31^ An accumulation of excess free cholesterol can lead to the development of cholesterol crystals in lipid droplets, which was associated with fibrosing NASH in a small human cohort.^32^ Further, free cholesterol could drive sterile inflammation by interacting with YAP-TAZ, which was also markedly increased in liver tissue of human NAFLD patients and murine livers of NAFLD models.^33–36^ Free cholesterol but not free fatty acids (FFAs) or triglycerides sensitized the liver towards the development of steatohepatitis induced by tumour necrosis factor (TNF) and fatty acid synthetase (FAS) in rodent models. This is due to depletion of mitochondrial glutathione,^37^ indicating an inflammation driving role of free cholesterol. Decreased glutathione levels can lead to augmented reactive oxygen stress (ROS) production and thereby to pro-inflammatory processes within the cell.^37^ Cholesterol is metabolized into bile acids (BAs), which are then secreted into the gut. In the gut, BAs act in the small intestine and play an important role in the uptake of cholesterol, fat, and vitamins (fat soluble). Primary BAs are metabolized by the intestinal microbiome to secondary BAs and also influence the constituency of the microbiome.^38,39^ In the terminal ileum, almost all BAs are actively reabsorbed.^38^ BA can act as signalling molecule through different receptors, such as Farnesoid X receptor (FXR) or the G protein–coupled bile acid receptor 1 (GPBAR1 also known as TGR5).^40^ Reduced hepatic fat accumulation as a result of reduced lipogenesis is observed after activation of FXR.^41^ Ileal FXR influences hepatic metabolism also through the production of fibroblast growth factor 15 (FGF15; FGF19 in humans) in the small intestine and subsequent increased oxidation of fatty acids and decreased hepatic lipogenesis.^40^ In mice, nor-ursodeoxycholic acid, by targeting mTORC1 in CD8+ T cells, ameliorated experimental cholestatic liver injury, indicating therapeutic mechanisms beyond hepatocyte metabolism^42^ (*Figure 1*).

#### 1.1.2 Dietary components affecting NAFLD

Besides overconsumption of calories and consecutive weight gain, fructose is a key player in the development and progression of NAFLD. Fructose is derived from the diet via sweetened beverages and processed food. Fructose increases lipogenesis by enhancing the available substrates for fatty acid synthesis via aldolase B and ketohexokinase action and also by activating transcription factors such as sterol regulatory element-binding protein 1c (SREBP1c) and others.^43^ A recent small study with paediatric and adolescents NAFLD patients described that the intakes of total calories, fat, and carbohydrates were similar between NAFLD and NASH patients; however, NASH patients had higher total intake of fructose, sugar, sucrose, and glucose.^44^ A meta-analysis including over 2000 individuals described that an excess of energy delivered by sugar-sweetened beverages (mainly by fructose) leads to an increase of liver fat.^45^

#### 1.1.3 Microbiome, the intestine, and NAFLD

A plethora of studies has underlined the importance of dysbiosis in the development of different stages of liver disease.^17,46–54^ These microbial changes were described on phylum, family, genus, and species level. For instance, *Proteobacteria* seem to be increased in NAFLD,^47–49,52^ whereas *Ruminococcaceae* or *Bifidobacteriaceae* were described to be decreased in NAFLD patients compared with healthy controls.^53,55^*Faecalibacterium prausnitzii*, a rather anti-inflammatory bacterial strain, is decreased in NAFLD patients,^55,56^ while *Robinsoniella* is an example for a genus to be increased in NAFLD.^52^

Two murine landmark studies from the early 2000s could show that the microbiome plays an essential role for the development of experimental NAFLD and body fat storage. One of these studies described that germfree mice that were colonized with cecal microbiome from conventionally raised mice showed an increase in body weight.^57^ Li *et al*.^58^ demonstrated that the probiotic VSL#3 protected against high-fat diet-induced liver damage in *ob/ob* mice. In the meantime, various mainly pre-clinical studies could prove that interference with the intestinal microbiome offers a possibility to influence the course of NAFLD and related diseases. Treatment with *Akkermansia muciniphila* did ameliorate liver disease, dyslipidaemia, and IR in different mouse models.^59,60^ In a double-blind randomized proof of concept study enrolling 40 overweight humans, pasteurized *A. muciniphila* improved insulin sensitivity and reduced plasma cholesterol.^61^ An important role of the microbiota in the development of liver disease was observed by the group of Friedman. They observed that the transfer of human microbiome, obtained from infants born to obese mothers 2 weeks after birth, into germfree mice induced hepatic inflammation and an increased susceptibility to inflammation and obesity induced by a western-style diet.^62^ In a recent study, Sookoian *et al*.^63^ showed a distinct microbial profile in liver tissue of NAFLD patients linked to obesity. Several strains could be associated with histologic inflammation. The liver microbiome is potentially populated from the gut and might shape the hepatic immune system^64^ (*Figure 1*).

Bacteria-derived metabolites can influence inflammatory and metabolic processes in the liver and other organs. The faecal metabolomic signature of NAFLD patients is altered when compared with healthy individuals. Some metabolic active substances are produced by bacterial enzymes out of the dietary components such as butyrate, propionate, and acetate, so-called short chain fatty acids (SCFAs). These metabolites are increased in the faeces of NAFLD patients^65^ and are bioactive agents, mainly by the binding to G protein–coupled receptors (GPCRs).^66^ However, it is not quite understood if SCFAs are drivers of disease progression in NAFLD or could also be beneficial as another recent study described an inverse relation between systemic SCFA levels and severity of liver disease in 74 cirrhosis patients.^67^ Moreover, SCFAs are mainly produced out of dietary fibres, and fibre intake was associated with a lower risk of mortality in chronic liver disease in a recently published cohort study using the NIH-AARP Diet and Health Study.^68^ Furthermore, it is believed that SCFAs have beneficial effects on obesity and obesity-related diseases.^69^ Another dietary-derived metabolite shown to play a role in NAFLD and related disease is trimethylamine-N-oxide (TMAO), which is produced in the liver out of trimethylamine (TMA). The intestinal microbiota are able to metabolize choline, carnitine, and phosphatidylcholine into TMA. A prospective study with 4007 participants could show a positive correlation between the baseline TMAO level and the risk for a major cardiovascular event (death, stroke, or myocardial infarction), which could be explained by an increased platelet activation through TMAO.^70,71^ Recently, it was demonstrated that TMAO was also measurable in faeces of mice fed a native starch diet^72^ as an earlier study could show that the genome of certain bacteria includes a TMA monooxygenase, which could indicate a non-hepatic source of systemic TMAO.^73^ Increased circulating TMAO levels were associated with the severity of NAFLD in a recently published study based on a case-control study with 60 NAFLD cases and 35 controls and a cross-sectional study with 1628 Chinese adults.^74^ A possible mechanistical explanation for the role of TMAO in metabolic diseases could be the activation of protein kinase R (PKR)–like endoplasmic reticulum kinase (PERK) through binding of TMAO.^75^ This is interesting as ER stress, which is partly coordinated by PERK, plays an important role in the development of NAFLD and related diseases such as T2D.^76,77^ A recent study with 307 healthy men from the Men’s Lifestyle Validation Study could identify microbial taxa such as *Alistipes shahii* being associated with TMAO concentrations.^78^

Another possible role for the gut in the development of NAFLD is via an increased intestinal permeability and thereby the translocation of bacteria and bacterial products via the portal vein into the liver and the systemic circulation. In a cross-sectional study using patients from the FLORINASH cohort, an increase in 16S rDNA concentration in patients with fibrosis was described in the discovery cohort comprising of 50 patients and the validation cohort with 71 patients.^79^ Schierwagen *et al*.^80^ described a systemic microbiome that seemed to be circulating in patients who received a transjugular intrahepatic portosystemic shunt (TIPS) procedure. Studies over 30 years ago had demonstrated endotoxaemia in patients with chronic liver disease as a surrogate marker of increased intestinal permeability.^81,82^ In a recent meta-analysis summarizing 14 studies with adult and paediatric patients, an increased intestinal permeability was shown in NAFLD patients compared with healthy controls.^83^ Lipopolysaccharide (LPS) induces nuclear factor of activated B-cell (NF-κB) activation through binding to toll-like receptor 4 (TLR4). In a study with 25 NASH and 25 simple steatosis patients, it was found that serum LPS levels were higher in the NASH cohort; additionally, there was a higher number of TLR4 expressing macrophages in liver biopsies of NAFLD patients compared with normal livers.^84^ An important role of TLR4 signalling in the development of liver disease has also mechanistically been described in mice studies, as TLR4-deficient mice are protected from experimental NAFLD and also alcohol-induced liver disease.^85–87^ The induction of TLR-4 in hepatic Kupffer cells (hKCs) leads to the production of pro-inflammatory cytokines enhancing hepatocyte dysfunction, necrosis, and apoptosis of hepatocytes and neutrophil recruitment into the liver. Moreover, hepatic stellate cells (HSCs) are activated by cytokines resulting in generation of extracellular matrix proteins leading to fibrosis/cirrhosis.^88^ hKC and HSC do also ‘communicate’ with each other, and this cross talk might be driving pro-inflammatory and fibrotic processes in the liver.^89^ This is partly also regulated by TLR4. LPS-dependent production of chemokines in HSC leads to the recruitment of hKC, which in turn produce transforming growth factor beta (TGFβ) and thereby activate HSC.^90^ Recently, MER proto-oncogene, tyrosine kinase (MerTK) was identified to play an important role in this cross talk, as its activation modulated the secreted proteins in macrophages and thereby promoted a pro-fibrogenic phenotype in human HSC in vitro.^91^

#### 1.1.4 Insulin resistance

NAFLD patients often also present with other features of the metabolic syndrome, and the liver is a central organ for metabolism, so a close relationship between NAFLD and IR is rather expected. IR is one of the main players in the pathophysiology of NAFLD as initially described by Marchesini *et al*.^92^ A rise of FFAs can induce hepatic IR in humans.^93^ Hepatic IR was associated with intrahepatic diacylglycerol (DAG) content in liver biopsies from obese, non-diabetic individuals.^94^ DAG content in the liver was correlated with protein kinase c epsilon-type (PKC-ε) activation.^94^ This axis was also described in a rodent model, where hepatic steatosis, induced by a short-term fat feeding, leads to activation of PKC-ε and c-Jun N-terminal kinases 1 (JNK1) and a possible interference with insulin receptor substrates 1 and 2 (IRS-1 and IRS-2) to the development of IR.^95^ An important driver of IR in the liver is inflammation, as mice expressing a constitutively active inhibitor of nuclear factor kappa-B kinase subunit beta (IKK-β) only in hepatocytes develop hepatic IR,^96^ while mice lacking IKK-β in hepatocytes are protected against hepatic IR after a high-fat diet, while they develop IR in muscle and fat.^97^ IKK-β is activated by oxidative stress,^98^ which is elevated in NAFLD patients.^99^ Further, IKK-β can also be activated by pro-inflammatory cytokines such as TNF, which are also elevated NAFLD patients.^100^ As NAFLD is extremely frequent in patients with T2D,^101,102^ IR seems also to be an attractive target for therapeutic modulation of NAFLD (see below) (*Figure 1*).

### 1.2 Clinical diagnosis of NAFLD

Today, besides liver biopsy, different non-invasive tests (NITs) can be used to diagnose NAFLD-like serum biomarkers, transient elastography (TE), and magnetic resonance elastography (MRE).

#### 1.2.1 Serum tests

**Figure 2:**
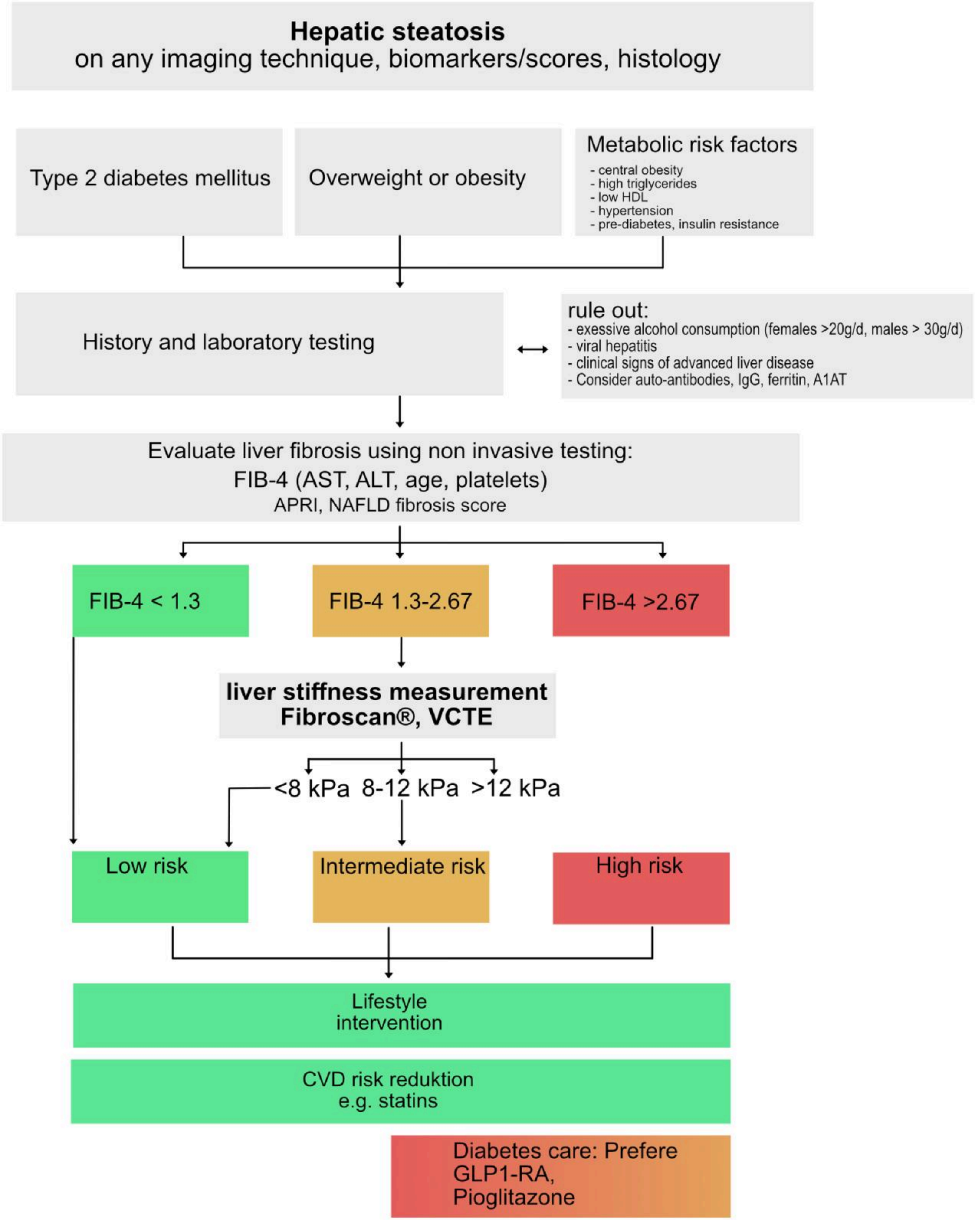
Diagnostic algorithm adapted from Kanwal *et al*.^106^ and the EASL CPG.^12^ A1AT, alpha 1 antitrypsin; ALT, alanin aminotransferase; AST, aspartat aminotransferase; CVD, cardio vascular disease; HDL-c, high-density lipoprotein cholesterol; IgG, immunoglobulin G; VCTE, vibration controlled transient elastography.

Serum tests comprise simple and inexpensive (non-patented) tests such as aspartat aminotransferase (AST)/alanin aminotransferase (ALT) ratio, AST to platelet ratio index (APRI), Fibrosis-4 (FIB-4), and NAFLD fibrosis score (NFS) compared with patented tests such as the FibroTest®, Fibrometer®, and Hepascore®. In a recent meta-analysis, Xiao *et al*.^103^ compared the performances of FIB-4, NFS, and APRI for the diagnoses of advanced fibrosis in NAFLD patients with summary AUROCS of 0.84, 0.84, and 0.77, respectively.^103^ The FIB-4 (age, AST, ALT, and platelet) can be used as a guidance to define patients who need further hepatic evaluation. Higher FIB-4 scores suggest advanced liver disease and a possible need for referral to a haepatologist. In patients with suspected NAFLD, a FIB-4 score < 1.3 rules out severe liver disease and no referral to a haepatologist or further diagnostic workup is needed. FIB-4 should be repeated in 1–3 years. In patients with suspected NAFLD and FIB-4 ≥ 1.3, TE for further workup is needed^104,105^ (*Figure 2*).

#### 1.2.2 Transient elastography

TE is a non-invasive tool for evaluating liver stiffness. It has a high applicability of >95% (in patients who are not morbidly obese), is easy to perform, and provides results in real time.^12,104^ Increased liver stiffness values are associated with liver fibrosis but can also occur in other conditions. However, liver stiffness is a physical property of the tissue, which not depends only on the amount of liver fibrosis but is also affected by inflammation, obstructive cholestasis, food ingestion, exercise, or venous congestion.^104^ TE enables evaluation of liver fibrosis in a broader population and is thereby feasible in view of NAFLD epidemic.^107^ Of note, TE should be repeated regularly also in patients already diagnosed with cirrhosis, as an increase in portal hypertension is a leading cause of cirrhosis-related complications.^108,109^

#### 1.2.3 Magnetic resonance elastography

MRE can usually be done on a regular MRI machine. Compared with liver biopsy and TE, MRE examines the whole liver, which makes results more robust. Another advantage is the higher applicability in difficult examination conditions like presence of ascites and obesity than TE. On the other side, MRE is costly and time-consuming.

#### 1.2.4 Biopsy

Liver biopsy is an invasive procedure with a mortality risk of ∼0.2%. Major bleedings occur in ∼0.6%.^110^ Today, liver biopsy is not universally needed to diagnose NAFLD because of the increasing benefit of NITs. Liver biopsy is indicated if NITs are discordant, to rule out other confounding liver diseases, defining stages of liver fibrosis or study purposes.^104,111^

### 1.3 NAFLD and CVD

Beside well-known liver-related mortality, CVD is a common cause for death in NAFLD patients. CVD-associated mortality in NAFLD patients increased by 14% from 2008 to 2018.^112^ Simon *et al*.^113^ showed recently increased overall mortality in all histological stages of NAFLD. Studies have proved the role of NAFLD in different cardiac disease manifestations like left ventricular dysfunction (LVD), atherosclerotic CV disease, and ischaemic heart disease. This suggests that NAFLD could be an independent predictor of CVD.^114,115^ In a recent meta-analysis including 34 043 patients with NAFLD, it was shown that NAFLD patients displayed an increased risk of both fatal and non-fatal CV events compared with non-NAFLD patients. Interestingly, this study furthered showed an increased risk of CV events in individuals with a greater severity of liver disease.^9,116^ Further, studies could show that especially hepatic fibrosis was associated with CVD and also liver-related outcome.^117,118^ Although the clinical association seems to be solid, a clear pathophysiological link between NAFLD and CVD is not established. It is rather thought to be a mixture between metabolic dysfunction, low-grade inflammation, dysregulated microbiome, and altered metabolism of (microbiome) derived products (e.g. TMAO; see above).^9^

### 1.4 Management of NAFLD

Although the prevalence of NAFLD and NASH is substantially increasing^5^ and is already a global burden, effective drug therapies are still missing. Cardiovascular disease and malignancies are the leading causes for death in patients with NAFLD.^119^ Therefore, the main treatment goal is to reduce CVD risk and malignancy risk as well as hepatic steatosis and inflammation. Here, we summarize some possible therapeutic strategies in NAFLD/NASH but will not discuss the importance of other CVD risk decreasing therapies such as statins, etc. Today, an increasing number of therapeutic options are available for the treatment of NAFLD. Important to note, most of them are not yet approved for the treatment of liver disease (Table 1).

**Table 1:** Available therapeutic options in NAFLD

	Liver enzymes	Steatosis	Inflammation	Fibrosis	Adverse events	Beneficial clinical aspects
GLP1 receptor agonists	+	+	+	+	Gastrointestinal	Weight loss, reduction of CV events
SGLT2 inhibitors	+	+	?	?	Genitourinary infections, dehydration	Reduction of CV events, nephron-protection, weight loss
Glitazones	+	+	+	+	Weight gain (mild), oedema, heart failure, bone fractures	reduction of CV events
Bariatric surgery	+	+	+	+	Invasive procedure, malnutrition	Weight loss

CV, cardio vascular; GLP1, glucagon-like peptide 1; SGLT2, sodium–glucose transporter 2.

#### 1.4.1 Probiotics

Multiple pre-clinical studies showed that the intestinal microbiota influences the course of NAFLD. Nevertheless, probiotics are not generally recommended for treating patients with NAFLD. Only few clinical studies tested probiotics in patients with NAFLD. VSL#3,^120^ different strains of *Lactobacilli*,^121^ and *Lactobacillus bulgaricus* and *Streptococcus thermophilus*^122^ were shown to be effective in reducing liver enzymes but not liver steatosis or fibrosis. More clinical trials are needed to define beneficial bacterial strains effective in NAFLD and NASH.

#### 1.4.2 Lifestyle change

An unhealthy lifestyle was associated early with NAFLD.^123^ Therefore, lifestyle change is mandatory in every patient with NAFLD. In a prospective study including 293 patients with biopsy-proven NASH, Vilar-Gomez *et al*.^124^ showed a reduction of hepatic steatosis and inflammation after a recommended lifestyle change within 12 months. Interestingly, the degree of weight loss was independently associated with the degree of NASH. All patients who achieved a weight loss ≥ 10% had a reduction of NAFLD Activity Score (NAS, histologic score), 90% had resolution of NASH, and 45% had regression of fibrosis.^124^ Sustained weight loss is challenging because it requires a transformation of behavioural patterns but depicts a cornerstone in NAFLD treatment. Generally, NAFLD patients are recommended to lose 7–10% of their body weight.^12^ Further, patients are advised to avoid alcohol consumption^12,125^ and high fructose intake.^126^ Physical activity was shown not only to reduce liver fat^127,128^ but also to reduce risk of CVD, obesity, and T2D.^129^ The European Association for the Study of the Liver (EASL) recommends over 150 min/week of moderate intensity physical activity (three to five sessions per week) combining aerobic and resistance training.^12,130^ Analysing data from 304 patients, Huber *et al*.^6^ could report a correlation between biopsy-proven lobular inflammation, diabetes, age, and sex with a lower health-related quality of life in NAFLD patients. The prospect for improvement of the quality of life could help to motivate patients to introduce and maintain the sometimes tedious changes in lifestyle.

#### 1.4.3 SGLT2 inhibitors

Sodium–glucose transporter 2 (SGLT2) inhibitors inhibit the SGLT2 transporter and promote urinary glucose excretion. Hereby, blood levels of glucose are decreased and IR can be improved in patients with T2DM.^131^ Kuchay *et al*. investigated the effect of empagliflozin on liver fat content in patients with T2D and NAFLD. Empagliflozin significantly reduced liver fat compared with controls (standard of care).^132^ In a meta-analysis including seven RTCs, the effect of SGLT2 inhibitors on NAFLD was investigated. Compared with placebo or reference therapy, empagliflozin, canagliflozin, or ipragliflozin showed a small improvement in liver fat content, assessed by ultrasound, FibroScan, and MRE. Furthermore, SGLT2 inhibitor treatment went along with a reduction of body weight (2–3 kg) and HbA1c reduction (0.8–1.0%). In all RCTs, SGLT2 inhibitors were associated with a reduction of transaminases,^133^ suggesting a possible amelioration of hepatic injury.

#### 1.4.4 Glucagon-like peptide 1 receptor agonists

Glucagon-like peptide 1 receptor agonists (GLP1-RAs) seem to exert the most promising beneficial effects on NAFLD or NASH. GLP1-RAs mimic the effects of physiological GLP1, e.g. stimulation of insulin secretion, inhibition of glucagon, gastrointestinal secretions, and motility. Furthermore, it reduces food intake by enhancing satiety.^134^ In a multi-centre placebo-controlled Phase 2 trial including obese patients with biopsy-proven NASH, liraglutide 1.8 mg/day for 48 weeks was effective to induce histological resolution of NASH and significantly improved histologic scores of NASH compared with those receiving placebo.^135^ Semaglutide 0.1, 0.2, or 0.4 mg was tested in patients with biopsy-confirmed NASH and liver fibrosis of Stages F1–F3. NASH resolution without worsening of fibrosis was achieved in 40% of the 0.1-mg group, 36% in the 0.2-mg group, 59% in the 0.4-mg group, and 17% in the placebo group. In the 0.4-mg group, the mean per cent weight loss was 13%.^136^ Important to note, liraglutide and other long-acting GLP1-RAs have been proved to reduce risk of adverse CVD and renal outcomes in patients with T2D.^137^

#### 1.4.5 BAs, BA metabolites, and FXR agonists

As also described above, BA signalling plays an important role in NAFLD development. FXR is a major regulator of BA metabolism and is involved in lipid and glucose metabolism.^138^ Different non-BA FXR agonists like tropifexor,^139^ cilofexor,^140^ and nidufexor^141,142^ have been tested in NAFLD and were proved to reduce liver fat content. Obeticholic acid (OCA), a modified BA and FXR agonist, was able to reduce fibrosis and histological features of liver disease in NASH patients.^143–145^ However, in one of these trials, an increase of very low-density lipoprotein (VLDL) and low-density lipoprotein cholesterol (LDL-c) particles and a decrease of HDL-c was observed during treatment but was reverted after discontinuation of the study drug, indicating a shift of lipoproteins from the liver to the systemic circulation.^146^ A Phase 2 dose finding study with 198 patients with NAFLD could show a reduction of ALT after 12 weeks of treatment with nor-ursodeoxycholic acid compared with placebo.^147^ In a 12-week, randomized, placebo-controlled study MET409, a non-BA agonist was shown to significantly reduce liver fat content compared with control group.^148^

#### 1.4.6 PPAR agonists

Peroxisome proliferator–activated receptors (PPARs) are transcription factors of nuclear hormone receptors with three subtypes PPAR-α, PPAR-γ, and PPAR-β/δ, which regulate lipid metabolism, energy homoeostasis, insulin sensitization, and glucose metabolism. Pioglitazone (PPAR-γ agonist) is a potent insulin sensitizer and is used in treatment of T2D. In a recent meta-analysis, pioglitazone was proved to be effective in reducing liver fibrosis and NASH.^149–151^ Interestingly, similar effects could be shown also in patients without T2D.^152^ Furthermore, pioglitazone displays protective effects on the vasculature, decreasing the risk of ischaemic stroke in patients with T2D or prediabetes.^153^ Pioglitazone lowers levels of triglyceride and LDL-c and increases HDL-c. Common side effects are weight gain, lower limb oedema as well as bone fractures, predominantly in post-menopausal women.^153^ The pan-PPAR agonist Lanifibranor is under clinical investigation in a phase III study. In a phase IIb study, Lanifibranor was effective in reducing NASH fibrosis, liver enzyme levels inflammatory, and fibrosis biomarkers.^154^

#### 1.4.7 Bariatric surgery

Weight loss is the cornerstone of NAFLD therapy,^155,156^ and the most potent therapy to induce weight loss is bariatric surgery.^157^ Although bariatric surgery is not a first-line therapy for NAFLD, it can be discussed for selected patients, especially if they qualify for surgery out of other reasons (co-morbidities or excessive obesity). An improvement of liver disease after bariatric surgery was described in many studies^157^; in a small clinical study from our clinic, we observed an improvement of liver histology after bariatric surgery, which was paralleled by an decrease of pro-inflammatory cytokines.^158,159^ A recent study from Germany described a histopathological resolution of NASH in 84% of observed patients 5 years after bariatric surgery, even in 45.5% of patients who had bridging fibrosis at baseline (i.e. a sign of advanced liver disease), fibrosis disappeared after 5 years in the follow-up biopsy.^160^ Together, these data indicate that bariatric surgery could be an appropriate therapeutic option for selected patients with NAFLD.

#### 1.4.8 Therapeutic options in the future

Thyroid hormones are involved in the regulation of hepatic triglyceride and cholesterol metabolism. Resmetirom, a thyroid receptor beta (TR-beta) agonist, was shown to improve liver steatosis^161^ and also lowered LDL-c and triglyceride concentration.^162^ FGF21 was proved to be effective in different animal models of obesity and NAFLD. The FGF21 variant LY2405319 was tested in patients with T2D mellitus. Here, LY2405319 improved lipid profiles and tended to decrease body weight, fasting insulin, and fasting glucose.^163^ Although more clinical data are needed, FGF21 could be an interesting therapeutic target in the future.

## 2. Conclusion

NAFLD is one of the major and relevant human diseases associated with an altered lifestyle with a rapidly growing incidence and prevalence in most countries of the world. NAFLD can cause a dramatic burden of disease throughout the different stages of chronic liver disease, including cirrhosis and associated complications such as HCC and decompensation (e.g. oesophageal variceal bleeding, ascites, and hepatic encephalopathy). Besides hepatic complications, the metabolic syndrome, T2D, and CVD are commonly observed in NAFLD patients. Although our understanding of the underlying mechanisms in the context of NAFLD is increasing, it is still not fully understood how NAFLD influences T2D and CVD on a pathophysiological level. One of the leading hypotheses is a sub-clinical pro-inflammatory environment arising from lipotoxicity, IR, and the intestinal microbiota. The main cornerstone in the treatment is lifestyle modification including weight loss, dietary intervention, and regular physical exercise. Although this is a difficult goal to obtain for many patients, it should be recommended and supported by the treating physicians. A range of therapeutic options, from conservative management and medical intervention towards operative procedures (e.g. bariatric surgery), has been developed with varying outcomes especially for the conservative treatment options. The pipeline of new therapeutic approaches is broad with some promising candidates. Taken together, NAFLD is a major healthcare issue that will rise in the future; thus, an increased awareness from physicians of different specialties is needed to tackle this disease.

## Data Availability

The data underlying this article will be shared on reasonable request to the corresponding author.
